# A Treatise on Therapeutics, Comprising Materia Medica and Toxicology, with Especial Reference to the Application of the Physiological Action of Drugs to Clinical Medicine

**Published:** 1883-07

**Authors:** 


**A Treatise on Therapeutics, Comprising Materia Medica and Toxicology, with Especial Reference to the Application of the Physiological Action of Drugs to Clinical Medicine.** By H. C. WOOD, M. D., Professor of Materia Medica and Therapeutics, and Clinical Professor of Diseases of the Nervous System, in the University of Pennsylvania; Physician to the Philadelphia Hospital, etc., etc. Fifth edition, revised and enlarged. Philadelphia: T. B. Lippincott & Co. 1883.

No one who has paid much attention to the study of therapeutics is ignorant of this leading text-book on the subject. The demand for the work seems to be on the increase, for the fourth edition was exhausted in about six months after publication. This fifth edition represents, more thoroughly than ever, the best therapeutic thought of the day and is fully up to the times. The book is open the to objection that too much space is occupied in giving the views of various experimenters whose results are often the most opposite; but though this may be a drawback to its use as a mere text-book for the student, it adds greatly to its value for the physicians really interested in the subject. One would like to read more of the author's own experiments, and less conservative expressions on many debatable subjects; but the book is not one to find fault with. In its especial field it is unequaled in any language, and no library is complete without it.

